# Study on the influence of syphilis on the outcome of frozen-thawed embryo transfer in infertility patients

**DOI:** 10.1016/j.heliyon.2024.e29342

**Published:** 2024-04-06

**Authors:** Lixia Miao, Lishuang Xu, Min Wang, Fang Xiong, Lian Zou, Yun Zhang, Meiling Weng, Huiming Zeng

**Affiliations:** Wuxi Maternity and Child Health Care Hospital, Affiliated Women’s Hospital of Jiangnan University, Wuxi, 214002, Jiangsu, China

**Keywords:** Syphilis infection, Frozen-thawed embryos, IVF-ET, Clinical results

## Abstract

**Objective:**

In this study, the effect of in vitro Fertilization-Embryo Transfer (IVF-ET) on the clinical outcome of patients with syphilis infertility during resuscitation cycle.

**Methods:**

A retrospective single-center method was adopted. This study included 4430 pairs of infertile patients who underwent syphilis detection. The influence of the syphilis freeze-thaw embryos transplantation outcome was studied in the patients with infertility by comparing the general clinical characteristics of patients (age, years of infertility, body mass index (BMI), basal follicle stimulating hormone (FSH), serum basal estradiol (Estradiol, E2), transplanted intimal thickness, the number of embryos transferred) and the clinical pregnancy (biochemical pregnancy rate, clinical pregnancy rate, implantation rate, live birth rate and abortion rate).

**Results:**

Firstly, in the clinical outcome of one frozen-thawed embryos transfer, the live birth rate of the woman's syphilis-infected group was lower than that of the uninfected group (71.3 % vs. 50.0 %), while the abortion rate was higher than that of the uninfected group (7.8 % vs. 26.7 %), and there was a statistical difference (*P* < 0.05), and there was no statistical difference in other indicators between other groups (*P* > 0.05). Secondly, in the clinical outcome of two frozen-thawed embryos transfers, the biochemical pregnancy rate (61.3 % vs. 28.6 %) and clinical pregnancy rate (42.9 % vs. 14.3 %) of the group which was infected with syphilis alone were lower than those of the uninfected group (*P* < 0.05), and other indicators among the other groups showed no statistical difference (*P* > 0.05). Thirdly, in the clinical outcomes of frozen-thawed embryos transfer three times or more, there was no significant difference in the clinical indicators between the syphilis infertility patients and the non-infected infertility patients (*P* > 0.05).

**Conclusion:**

When the syphilis infertility patients and the non-infected infertile patients underwent IVF-ET treatment for the first time, the live birth rate and abortion rate of the syphilis group were significantly different (*P* < 0.05). In the outcome of two transplants, the biochemical pregnancy rate and clinical Pregnancy rates were significantly reduced so patients with syphilis infertility who undergo IVF-ET should be informed about the risk of adverse clinical outcomes.

## Highlights

• The live birth rate and abortion rate of the syphilis group were significantly different.
• The biochemical pregnancy rate and clinical pregnancy rate of the syphilis group were lower than that of the uninfected infertile patients, which suggested that syphilis has a certain correlation with repeated implantation failure in infertile patients.
• Patients with syphilis infertility who undergo IVF-ET should be informed about the risk of adverse clinical outcomes.

## 1 Introduction

Syphilis is a chronic sexually transmitted disease caused by *Treponema Pallidum* (TP), which is mainly transmitted through sexual, blood-borne and mother-to-child transmission [1]. There were approximately 7 million new syphilis infections worldwide in 2020 and an estimated 1 million pregnant women infected with syphilis in 2016, resulting in 350,000 infants infected with syphilis, resulting in adverse birth outcomes, including 200,000 stillbirths and neonatal deaths, and a range of clinical manifestations that can persist for years if left untreated, most commonly infertility [2]. Male syphilis infection leads to reproductive tract diseases, which can lead to inflammation in mild cases and infertility in severe cases [3]. In women, mild syphilis infection can lead to endometritis, and severe syphilis can cause fallopian tube blockage and infertility [4]. As a result, In Vitro Fertilization (IVF) has become a last resort to treat infertile couples [5,6]. Since the first reported successful conception of a Frozen Embryo Transfer (ET) in 1983, ET technology has been widely used in the clinic. ET technology can reduce the risk of Ovarian Hyper-stimulation Syndrome (OHSS) and reduce the incidence of polypeptide pregnancy [7]. Many studies have also confirmed that the pregnancy outcome of ET is not worse than that after fresh embryo transfer [8], and the pregnancy outcome of ET is better than that after fresh embryo transfer even in women with high ovarian response [9]. The relationship between syphilis and infertility has been partially reported. Lin et al. [10,11] found that patients with a history of syphilis infection had lower embryonic quality development potential when undergoing IVF-ET treatment. Zádori et al. showed that whether a patient was infected with syphilis did not affect the clinical pregnancy outcome of IVF-ET, while Cory et al. showed that syphilis infection could reduce the clinical pregnancy rate and embryo implantation rate in IVF-ET treatment, but there was no statistical difference [12,13]. All the existing studies have focused on the effects of fresh cycle syphilis on embryo quality and clinical pregnancy outcomes, and few scholars have studied the clinical outcomes of repeated freeze-thaw embryo transfer in syphilis infected patients after IVF-ET treatment.

Therefore, in this study, the clinical indicators of resuscitation cycle were retrospectively analyzed in a single center to investigate the influence of syphilis on clinical outcomes in infertility patients undergoing IVF-ET treatment with one transplant, two transplants and three transplants or more freeze-thaw embryo transfers.

## 2 Data and methods

### 2.1 Research object

**Fig. 1:**
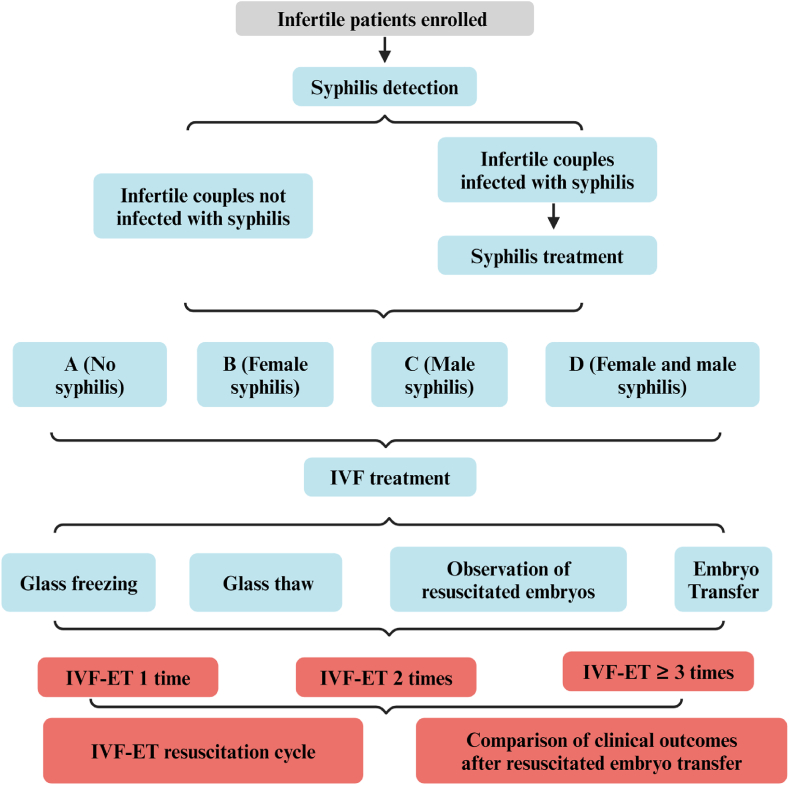
The overall experimental flow of IVT-ET.

The whole study was approved by the Medical Ethics Committee of Wuxi Maternal and Child Health Hospital (2023-06-1213-67). A retrospective single-center study was conducted to select infertile couples who underwent syphilis detection before receiving IVF-ET treatment at the Reproductive Medicine Center of Wuxi Maternal and Child Health Hospital from January 2013 to April 2021. All syphilis patients have received formal treatment before receiving IVF treatment. The whole study was shown in Fig. 1.

Inclusion criteria: (1) IVF-ET treatment from January 2013 to April 2021; (2) Age ≤36 years old [14]; (3) Resuscitation cycles of 1 transplant, 2 transplant and 3 transplant; (4) Syphilis test before IVF-ET; (5) The embryos of patients with resuscitation transplantation cycle were all transferable and above grade embryos.

Exclusion criteria: (1) Uterine malformation or uterine dysplasia; Hepatitis B, hepatitis C, AIDS, mycoplasma genitalium and chlamydia infection. (3) Resuscitated embryos are not available for transplantation; (4) Data incomplete cycle; (5) Lost data; (6) Patients with endometrial thickness <8.

### 2.2 Grouping basis and number of inclusion cycles

A total of 4430 pairs of infertile patients participated in the experiment. A total of 1940 resuscitation cycles were included in the first freeze-thaw embryo transfer, 1444 resuscitation cycles were included in the patients with 2 transplants, and 1046 resuscitation cycles were included in the patients with ≥3 transplants. According to whether the infertile patients were infected with syphilis were divided into four groups, group A was non-infected with syphilis (the control group), group B was infected with syphilis by a single woman, group C was infected with syphilis by a single man, and group D was infected with syphilis by both men and women. According to the number of transplants, they are divided into A1, B1, C1, D1(1 is 1 transplant); A2, B2, C2, D2 (2 represents two migrations); A3, B3, C3, D3 (3 was ≥3 transplantation times), and the number of cycles in each group was: A1: n = 1909 cases, respectively. B1: n = 22 cases; C1: n = 4 cases; D1: n = 5 cases. A2: n = 1420 cases; B2: n = 14 cases; C2: n = 10 cases; D2: n = 0 example. A3: n = 1020 cases; B3: n = 23 cases; C3: n = 3 cases; D3: n = 0 example.

### 2.3 Embryo scoring criteria

At present, the clinical embryo morphological quality evaluation mainly refers to the Istanbul Consensus published in 2011.

According to consensus and the work of the Center [15], day 3 embryo were divided into 4 grades: Grade I embryos had 7 to 10 blastomes with uniform blastomes size, uniform cytoplasm and fragments ≤5 %; Grade II embryos had <10 or >6 blastomes, uniform in size; Or debris 5 %–10 %; Or uneven blastomere size; The size of blastomes of grade III embryos is uneven, the shape is poor, and the fragments are less than 10 %. Or debris 10 %–20 %; Or 4–5 blastomere embryos without fragmentation, uniform size; The size of the blastomes of grade IV embryos was severely uneven, with fragments >20 %. The Center stipulates that Grade I to III embryos should be cryopreserved or continued blastocyst culture after the informed consent of the patient, and grade IV embryos should be continued blastocyst culture or discarded.

The scoring of day 5/6 embryo was performed using Gardner scores. They are scored mainly according to the size of the blastocyst cavity, the inner cell mass, and the presence of trophoblast cells. Day 5/6 embryo with a grade of 4BCE and above were vitrified and frozen.

### 2.4 Glass freezing

Equilibration Solution (ES) was made into microliter droplets, left at room temperature for 15 min, and then transferred into 1 to 2 embryos. Equilibration of day 3 embryo (10–12 min) and day 5/6 embryo (12–15 min) was transferred. The embryos in ES were transferred to Vitrification Solution (VS), and the embryos were quickly transferred to the marked position at the top of the frozen load rod with very small droplets within 1 min, and the load rod was immediately put into liquid nitrogen and filled with tubes for preservation.

### 2.5 Glass thaw

Patients with Thawing plans to be thawed were taken out of the liquid nitrogen tank and the head of the rods with embryos were immediately immersed in pre-heated and balanced Thawing Solution (TS) at 37 °C for 1 min. Then the thawed embryos were transferred to Diluent Solution (DS) separately stood for 3 min, Washing Solution l (WS1) for 5min and Washing Solution 2 (WS2) for 3 min, and finally transferred the embryos into G2 (Vitrolife) culture medium. The culture was continued for 2 h in an incubator at 37 °C and 6 % CO_2_ to prepare for transplantation.

### 2.6 Observation of resuscitated embryos

Cleavage embryo: If the embryo blastomere is not degraded, uniform or the embryo blastomere is degraded and the survival of all blastomere is >50 % and shiny, the blastomere is uniform before transplantation. Blastocyst: The blastocyst cavity has some expansion.

### 2.7 Embryo transfer

Patients who did not undergo transplantation during the fresh cycle or did not become pregnant after fresh transplantation on the third day after egg retrieval were selected, and the available remaining embryos were cryopreserved by vitrification and then resuscitated at the appropriate time. The recovery cycle of the center was prepared according to the natural cycle, hormone replacement cycle and artificial cycle, and progesterone intramural injection was started when the endometrial thickness was ≥8 mm, and frozen embryos were transferred from 3 to 5 days. Day 3 embryo and day 5/6 embryo were routinely transferred, and the number of embryos was controlled to 1 or 2.

### 2.8 Determination of pregnancy outcome

After 12 days of embryo transplantation, a positive morning urine pregnancy test and blood HCG ≥10 m IU/mL were identified as biochemical pregnancy. After 28 days of embryo transfer, if the echo of pregnancy sac, germ or fetal cardiac tube pulsation are found by B-ultrasound examination, it is judged as clinical pregnancy.

### 2.9 Observation indicators

(1) hCG positive rate in resuscitation transplant cycle (biochemical pregnancy rate) = hCG positive cycle number/resuscitation transplant cycle number × 100 %; (2) Clinical pregnancy rate of resuscitation transplant cycle: clinical pregnancy rate = number of intrauterine and extrauterine pregnancy cycles/number of transplant cycles × 100 %; (3) Abortion rate = number of abortion cycles/number of clinical pregnancy cycles × 100 %; (4) Implantation rate of resuscitation cycle = number of pregnancy capsules/total transplanted embryos × 100 % (number of pregnancy capsules for single embryo transfer is only 1); (5) Live birth rate = number of live birth cycles/transplant cycles × 100 %.

### 2.10 Syphilis test

All serum samples are first screened for syphilis using a non-syphilis RPR test, followed by a TPPA test to confirm a syphile-positive sample.

### 2.11 Statistical methods

SPSS 23.0 software was used for statistical analysis of quantitative data. First, normality test was performed. If the normal distribution was in line, the mean ± standard deviation (x ± s) was used to indicate the difference between groups, and T-test was used to analyze the difference between groups. If the distribution did not conform to normal, the median and quartile [M (P25, P75)] were used, and the Mann-Whitney *U* test analyzed the difference between groups. Trend Chi-square test or Fisher exact probability method were used to analyze the qualitative data. All the results showed significant difference with *P* < 0.05.

## 3 Results

### 3.1 IVF-ET resuscitation cycle

Comparison of general clinical basic data: Patients in groups B1, C1 and D1 were compared with those in group A1 in terms of age, infertility years, Body Mass Index (BMI), Follicle Stimulating Hormone (FSH) and serum Estradiol, respectively. E2), endometrial thickness, and number of transplanted embryos were not statistically significant (*P* > 0.05), and the results were shown in Table 1. Similarly, there was no statistical difference in the above indexes between groups B2 and C2 and A2 respectively (*P* > 0.05), and the results were shown in Table 3. There was no significant difference in the above indexes between B3 and C3 groups and A3 groups (*P* > 0.05), and the results were shown in Table 5.

**Table 1:** Basic clinical data of IVF-ET patients of 1 time freeze-thaw embryo transfer.

Groups	A1	B1	z	*P* (A1 and B1)	C1	z	*P* (A1 and C1)	D1	z	*P* (A1 and D1)
Age (years old)	30.0 (28.0, 32.0)^a^	32.0 (27.75, 36.0)^a^	−1.924	0.054	31.0 (27.0, 32.8)^a^	−0.206	0.837a	29.0 (27.0, 31.0)^a^	−0.804	0.421
Infertility years (years)	3.67 (2.33, 5.42)^a^	3.0 (1.8, 6.3)^a^	−0.872	0.383	1.0 (0.9, 5.7)^b^	−1.701	0.089	2.67 (0.79, 4.5)^a^	−1.461	0.144
BMI (kg/m2)	21.83 (20.1, 24.0)^a^	23.3 (21.5, 26.1)^a^	−1.963	0.05	19.9 (19.3, 20.2)^b^	−1.978	0.05	22.0 (21.1, 23.8)^a^	−0.425	0.671
Basic FSH (mIU/mL)	6.50 (5.45, 7.82)^b^	7.4 (5.2, 10.6)^a^	−1.937	0.053	6.9 (5.9, 7.97)^a^	−0.462	0.644	5.99 (4.9, 7.2)^c^	−0.932	0.351
Base E2 (pg/mL)	37.42 (27.8, 49.96)^b^	38.1 (28.98, 52.3)^b^	−0.747	0.455	47.1 (27.7, 122.0)^a^	−0.738	0.461	44.5 (27.9, 47.8)^a^	−0,185	0.853
Transplantation intimal thickness (mm)	9.2 (8.5,10.5)^a^	9.0 (8.4, 9.3)^a^	−1.478	0.139	9.75 (9.4, 13.2)^a^	−1.348	0.178	9.2 (8.3, 1.65)^a^	−0.238	0.812
Transplants (pieces)	2.00 (1.00,2.00)^a^	2.00 (1.75, 2.00)^a^	−0.98	0.327	2.0 (1.25, 2.0)^a^	−1.207	0.227	1.0, (1.00, 1.5)^b^	−1.351	0.177

The median and quartile [M (P25, P75)] were used, and the Mann-Whitney *U* test were analyzed among groups. Values with different superscript letters within each column are significantly different (*P* < 0.05).

**Table 2:** The results of 1 time freeze-thaw embryo transfer [% (n/n)].

Groups	A1	B1	χ2	*P* (A1 and B1)	C1	χ2	*P* (A1 and C1)	D1	χ2	*P* (A1 and D1)
Biochemical pregnancy rate	82.9 % (1582/1909)	77.3 % (17/22)	–	0.567^b^	75.0 % (3/4)	–	0.529^b^	80.0 % (4/5)	–	1.000^b^
Clinical pregnancy rate	77.7 % (1484/1909)	68.2 % (15/22)	–	0.303^a^	75.0 % (3/4)	–	1.000^b^	80.0 % (4/5)	–	1.000^b^
Implantation rate of resuscitation cycle	59.0 % (1924/3263)	50.0 % (20/40)	1.311	0.252^a^	50.0 % (4/8)	–	0.724^a^	83.3 % (5/6)	–	0.411^b^
Live birth rate (carrying baby home rate)	71.3 % (1362/1909)	50.0 % (11/22)	4.823	0.034^a^	75.0 % (3/4)	–	1.000^b^	80.0 % (4/5)	–	1.000^b^
Abortion rate	7.8 % (116/1484)	26.7 % (4/15)	–	0.027^b^	0.0 % (0/3）	–	1.000^b^	0.0 % (0/4)	–	1.000^b^

^b^ was Fisher exact probability method, *P* < 0.05 showed a statistical difference..

a was the trend Chi-square test.

**Table 3:** Basic clinical data of IVF-ET patients of 2 times freeze-thaw embryo transfer.

Groups	A2	B2	z	*P* (A2 and B2)	C2	z	*P* (A2 and C2)
Age (years old)	30.0 (28.0, 32.0)^a^	29.0 (29.0, 33.0)^a^	−0.372	0.71	31.0 (28.75, 31.25)^a^	−0.302	0.763
Infertility years (years)	4.00 (2.50, 5.83)^a^	2.9 (2.2.4.1)^b^	−1.621	0.105	4.0 (3.2, 5.21)^a^	−0.091	0.928
BMI (kg/m^2^)	21.83 (20.3, 24.0)^a^	22.8 (20.7, 25.2)^a^	−0.653	0.514	22.04 (20.05, 24.0)^a^	−0.144	0.886
Basic FSH (mIU/mL)	6.49 (5.46, 7.78)^a^	6.4 (5.1, 10.78)^a^	−0.804	0.422	5.75 (5.52, 7.0)^b^	−0.838	0.402
Base E2 (pg/mL)	38.2 (28.33, 50.2)^a^	33.0 (26.7, 44.0)^b^	−0.999	0.318	40.9 (35.58, 51.0)^a^	−1.034	0.301
Transplantation intimal thickness (mm)	9.00 (8.40, 10.1)^a^	10.45 (9.8, 11.1)^a^	−1.963	0.05	8.45 (8.2, 10.7)^a^	−1.073	0.283
Transplants (pieces)	2.00 (1.00, 2.00)^a^	1.0 (1.00, 2.00)^b^	−1.795	0.073	1.5 (1.00, 2.00)^a^	−1.072	0.284

The median and quartile [M (P25, P75)] were used, and the Mann-Whitney *U* test were analyzed among groups. Values with different superscript letters within each column are significantly different (*P* < 0.05).

### 3.2 Comparison of clinical outcomes after resuscitated embryo transfer

**Fig. 2:**
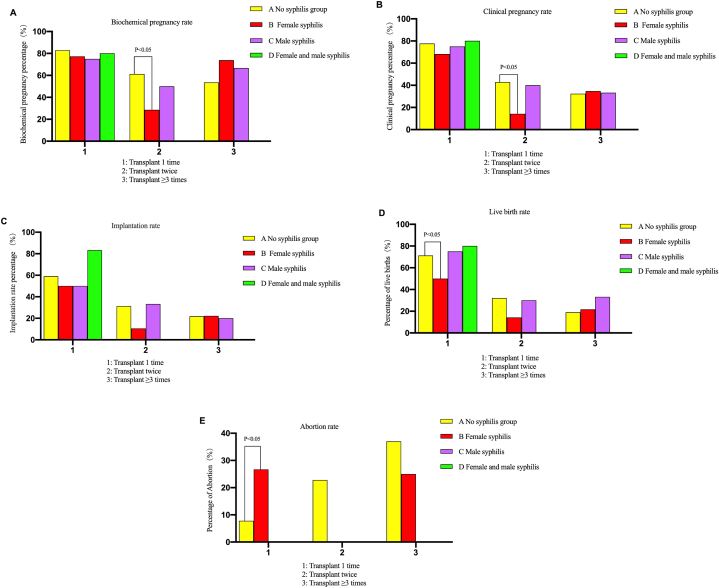
The results of Biochemical pregnancy rate Biochemical pregnancy rate, Clinical pregnancy rate, Planting rate, Live birth rate (carrying baby home rate) and Abortion rate. Only the results with statistical significance were marked in the bar chart, and no *P* < 0.05 indicated that there was no statistical difference between the groups, that is, *P* > 0.05.

The live birth rate in group B1 was lower than that in group A1 (71.3 % vs. 50.0 %), and the abortion rate was higher (7.8 % vs. 26.7 %), and the difference was statistically significant (*P* < 0.05). There were no significant differences in biochemical pregnancy rate, clinical pregnancy rate and implantation rate among all groups (*P* > 0.05), and there were no statistically significant differences in various indexes between groups C1 and D1 compared with group A1 (*P* > 0.05). The results could be seen in Table 2 and Fig. 2 (A – E).

After two transplants, the biochemical pregnancy rate and clinical pregnancy rate in group B2 were lower than those in group A2 (61.3 % vs. 28.6 % and 42.9 % vs. 14.3 %, *P* < 0.05), while the implantation rate, live birth rate and abortion rate were not significantly different from those in group A2 (*P* > 0.05). There was no statistical difference in clinical indicators between group C2 and group A2 (*P* > 0.05), and the results were shown in Table 4 and Fig. 2 (A – E).

**Table 4:** The results of 2 times freeze-thaw embryo transfer [% (n/n)].

Groups	A2	B2	χ2	*P* (A2 and B2)	C2	χ2	*P* (A2 and C2)
Biochemical pregnancy rate	61.3 % (870/1420)	28.6 % (4/14)	6.227	0.024^a^	50.0 % (5/10)	–	0.523^b^
Clinical pregnancy rate	42.9 % (609/1420)	14.3 % (2/14)	4.638	0.032^a^	40.0 % (4/10)	–	1.000^b^
Implantation rate of resuscitation cycle	31.4 % (749/2384)	10.5 % (2/19)	3.829	0.078^a^	33.3 % (5/15)	–	1.000^b^
Live birth rate (carrying baby home rate)	32.1 % (456/1420)	14.3 % (2/14)	–	0.248^b^	30.0 % (3/10)	–	1.000^b^
Abortion rate	22.8 % (139/609)	0.0 % (0/2)	–	1.000^b^	0.0 % (0/4)	–	0.579^b^

^b^ was Fisher exact probability method, *P* < 0.05 showed a statistical difference.

a was the trend Chi-square test.

**Table 5:** Basic clinical data of IVF-ET patients of ≥3 times freeze-thaw embryo transfer.

Groups	A3	B3	z	*P* (A3 and B3)	C3	z	*P* (A3 and C3)
Age (years old)	30.0 (28.0, 32.0)^a^	31.0 (27.0, 34.0)^a^	−1.444	0.149	30.0 (30.0, 30.0)^a^	−0.08	0.937
Infertility years (years)	4.00 (2.50, 6.00)^b^	4.0 (3.08, 7.75)^b^	−1.031	0.302	5.75 (5.50, -)^a^	−1.356	0.175
BMI (kg/m^2^)	21.5 (19.5, 23.8)^a^	22.15, (20.8, 24.0)^a^	−1.571	0.116	21.2 (21.2, 21.2)^a^	−0.346	0.729
Basic FSH (mIU/mL)	6.28 (5.19, 7.52)^c^	7.11 (6.07, 7.92)^b^	−1.619	0.105	9.37 (9.37, -)^a^	−2.56	0.01
Base E2 (pg/mL)	35.82 (27.0, 47.4)^b^	47.38 (30.85, 56.2)^a^	−1.665	0.096	38.4 (32.0, -)^a^	−0.014	0.989
Transplantation intimal thickness (mm)	9.00 (8.40, 10.3)^b^	8.9 (8.3, 10.3)^b^	−0.232	0.817	12.4 (10.4, -)^a^	−2.249	0.024
Transplants (pieces)	2.00 (1.00, 2.00)^a^	2.00 (1.00, 2.00)^a^	−0.755	0.45	2.00 (1.00, -)^a^	−0.063	0.949

The median and quartile [M (P25, P75)] were used, and the Mann-Whitney *U* test were analyzed among groups. Values with different superscript letters within each column are significantly different (*P* < 0.05).

There were no significant differences in biochemical pregnancy rate, clinical pregnancy rate, implantation rate, live birth rate and abortion rate in B3 and C3 groups compared with A3 groups (*P* > 0.05). The results were shown in Table 6 and Fig. 2 (A – E).

**Table 6:** The results of ≥3 times freeze-thaw embryo transfers [%(n/n)].

Groups	A3	B3	χ2	*P* (A3 and B3)	C3	χ2	*P* (A3 and C3)
Biochemical pregnancy rate	53.6 % (547/1020)	73.9 % (17/23)	3.727	0.054^a^	66.7 % (2/3)	–	1.000^b^
Clinical pregnancy rate	32.4 % (330/1020)	34.8 % (8/23)	0.061	0.806^a^	33.3 % (1/3)	–	1.000^b^
Implantation rate of resuscitation cycle	21.9 % (371/1694)	22.2 % (8/36)	0.002	0.963^a^	20.0 % (1/5)	–	1.000^b^
Live birth rate (carrying baby home rate)	19.0 % (194/1020)	21.7 % (5/23)	–	0.788^b^	33.3 % (1/3)	–	0.470^b^
Abortion rate	37.0 % (122/330)	25.0 % (2/8)	–	0.715^b^	0.0 % (0/1)	–	1.000^b^

^b^ was Fisher exact probability method, *P* < 0.05 showed a statistical difference..

a was the trend Chi-square test.

## 4 Discussion

Syphilis is mainly spread through maternal-neonatal transmission, blood transfusion and sexual intercourse, which a sexually transmitted disease (STD) induced by Treponema pallidum [16]. The incidence rate of syphilis has risen rapidly in China since 1978, and it has become the most common STD in economically developed regions [17]. Syphilis infectionof the reproductive tract can cause inflammatory disease and infertility. Reproductive tract infection and inflammation cause 8 % and 35 % of all cases of male infertility, respectively [18]. In women, it can result in fallopian tube obstruction and endometritis. Approximately 72.4 million people worldwide have infertility problems. In vitro fertilization (IVF) may be the last resort for couples attempting to overcome infertility [19]. The relationship between syphilis and infertility has been rarely reported in the literature.

Age is related to female fertility. With the increase of age, fertility tends to decline, especially from the age of 37, female fertility declines more rapidly [14]. Therefore, the object of this study is female infertility patients ≤36 years old. Other chronic infectious diseases (HBV, HCV, and HIV) also have a certain impact on IVF-ET outcomes. Therefore, in this study, the age of infertility patients in the uninfected and infected groups was matched, and the confounding influence of other chronic infectious diseases such as mycoplasma genitalium and chlamydia was also excluded.

The results of this study show that: (1) In the clinical outcome of one transplant, compared with the non-syphile-infected group, the live birth rate in the resuscitation cycle of female syphile-infected patients decreased and the abortion rate increased, with statistical significance (*P* < 0.05). Cory et al. found that the clinical pregnancy rate and implantation rate in the female syphilis group decreased compared with the non-syphile-infected group, but there was no statistical difference [13]. Zádori et al. found no statistical difference in clinical pregnancy rate and implantation rate between the syphilis group and the control group [12]. The reasons for the controversy might be as follows: (1) The research objects were different: this study was the result of frozen embryo transfer in resuscitation cycle, while the research object of Cory et al. and Zádori et al. was the result of fresh cycle embryo transfer; (2) Different types of embryos: frozen embryos in this study included frozen day 3 embryo and day 5/6 embryo, while fresh cycle research only included day 3 embryo. (3) Differences in age of patients: in order to exclude the influence of age confounding factors, the object of this study is infertility patients ≤36 years old. (4) Existing studies have not divided the number of authors' transplants purely. Therefore, the difference in clinical pregnancy rate and abortion rate found in the syphile-infected group compared with the non-syphile-infected group in this paper was not inconsistent with existing reports, and there were also some literature reports that syphilis could cause abortion [20], which could support the results of this study. (1) In the outcome of the first transplantation, there were no statistical differences in the biochemical pregnancy rate, clinical pregnancy rate, resuscitation cycle implantation rate, live birth rate and abortion rate in the female syphilis positive group, the male syphilis positive group and both male and female syphilis positive group compared with the non-syphilis infected group (*P* > 0.05), but the implantation rate in the male syphilis group showed a decreasing trend. The results were consistent with those of Lin et al. [10] who found that the embryonic development potential and implantation rate of male syphilis patients were reduced after IVF-ET treatment. However, studies on syphilis infertility patients still need to continue to accumulate large sample size to further confirm the existing studies. (2) Compared with the uninfected group, the biochemical pregnancy rate and clinical pregnancy rate in the syphile-positive group had a decreased trend (*P* < 0.05), while the implantation rate, live birth rate and abortion rate had no statistical difference; There was no statistical difference in clinical indicators between the male syphilis positive group and the male and female both syphilis positive group and the non-syphilis infected group. These results of no statistical difference should be further accumulated clinical samples for correction. (3) Compared with the non-infected group, there was no statistical difference in various clinical indicators between the different groups of syphilis patients with ≥3 transplants (*P* > 0.05). The possible reason is that confounding factors such as intima thickness and small sample size in the syphilis group may lead to bias in the results, which requires further expanding the sample size for follow-up research and correction. In conclusion, there was no statistical difference in clinical outcome among the results of one transplant, while the biochemical pregnancy rate and clinical pregnancy rate decreased between the results of two transplants, suggesting that syphilis was somewhat correlated with repeated implantation failure in infertile patients. The statistical difference between the three transplants or more and the results above may be caused by the small number of cases. It is also necessary to continue to accumulate the number of syphilis patients and increase the number of multi-center studies to further explore. The limitation of this study is that the sample size of the syphilis group is relatively small, but the “imbalance” of the sample size of the control group and the syphilis group more truly reflects the distribution of syphilis patients in infertility patients. If the proportion of the experimental group and the control group is deliberately controlled at 1:1, statistical analysis can be more accurate, but this proportion is a virtual statistical result, which does not have extrapolation in clinical practice. However, the focus of future research should be to accumulate more large sample numbers, or combine with further multi-center studies to further clarify whether subgroup parameters are influential factors for the clinical outcome of IVF-ET treatment for syphilis infertility patients during resuscitation cycle, and provide theoretical guidance for the clinical outcome of subsequent syphilis infection patients undergoing IVF-ET treatment during resuscitation cycle.

Syphilis (as an STD) can result in endometrial damage, which can induce pelvic inflammatory diseases [21]. Moreover, this injury leads to the endometrial lining thickening, abnormal endometrial functioning endometrial receptivity reducing, resulting in infertility. It also leads to repeated repair and regeneration processes [22]. However, compared with the control group, the numbers of normally cleaved oocytes and normally fertilized oocytes as well as the implantation rate were significantly lower for the syphilis group, and these findings may be related to pelvic inflammation, which may reduce the implantation rate (12 versus 33 %) [23].

Syphilis can cause pelvic inflammatory disease in women, and can also regenerate the endometrium, resulting in reduced receptivity of the endometrium, and thus infertility [24]. However, most patients do not show obvious clinical symptoms, resulting in many patients do not know whether they are infected with syphilis, so people with multiple sexual partners should be screened at least once a year to avoid delaying the best time for treatment. The medical staff should do a good job in education and psychological counseling when the syphilis infertile patients undergo IVF-ET treatment, and inform the patients that syphilis infection may affect the clinical outcome of IVF-ET, and allow the patients to fully informed consent before IVF-ET treatment is feasible. If there are available embryos left in patients with syphilis that need to be cryopreserved, enclosed cryopreserved embryos should be used as far as possible to avoid iatrogenic cross infection [1].

In summary, for syphile-infected infertile patients ≤36 years old who undergo the one or twice freeze-thaw embryo transfer for IVF-ET treatment, inform them that their clinical outcome may be affected by syphilis, and let them be fully informed before carrying out relevant treatment; Further accumulate the relevant data of syphile-infected infertile patients ≤36 years old, further track the influence of their clinical outcomes and correct. At the same time, we should further accumulate syphilis-related data to track the influence factors of syphilis on the offspring of infected patients.

## Data availability statement

Data will be made available on request.

## Ethics statement

This study was conducted after applying to and receiving approval from the Medical Ethics Committee of Wuxi Maternal and Child Health Hospital (2023-06-1213-67).

## CRediT authorship contribution statement

**Lixia Miao:** Writing – original draft. **Lishuang Xu:** Data curation. **Min Wang:** Formal analysis. **Fang Xiong:** Investigation. **Lian Zou:** Validation. **Yun Zhang:** Data curation. **Meiling Weng:** Formal analysis, Data curation. **Huiming Zeng:** Supervision.

## Declaration of competing interest

The authors declare that they have no known competing financial interests or personal relationships that could have appeared to influence the work reported in this paper.
